# Effects of Robot Animacy and Emotional Expressions on Perspective-Taking Abilities: A Comparative Study across Age Groups

**DOI:** 10.3390/bs13090728

**Published:** 2023-08-31

**Authors:** Xucong Hu, Song Tong

**Affiliations:** 1 Faculty of Psychology, Southwest University, Chongqing 400715, China; huxucong@email.swu.edu.cn; 2 Department of Psychology, Tsinghua University, Beijing 100084, China

**Keywords:** aging population, companion robots, facial animacy, cognitive empathy

## Abstract

The global population is inevitably aging due to increased life expectancy and declining birth rates, leading to an amplified demand for innovative social and healthcare services. One promising avenue is the introduction of companion robots. These robots are designed to provide physical assistance as well as emotional support and companionship, necessitating effective human–robot interaction (HRI). This study explores the role of cognitive empathy within HRI, focusing on the influence of robot facial animacy and emotional expressions on perspective-taking abilities—a key aspect of cognitive empathy—across different age groups. To this end, a director task involving 60 participants (30 young and 30 older adults) with varying degrees of robot facial animacy (0%, 50%, 100%) and emotional expressions (happy, neutral) was conducted. The results revealed that older adults displayed enhanced perspective-taking with higher animacy faces. Interestingly, while happiness on high-animacy faces improved perspective-taking, the same expression on low-animacy faces reduced it. These findings highlight the importance of considering facial animacy and emotional expressions in designing companion robots for older adults to optimize user engagement and acceptance. The study’s implications are pertinent to the design and development of socially effective service robots, particularly for the aging population.

## 1. Introduction

The 21st century is witnessing a demographic shift as the global population ages, pressuring global social and public healthcare services [1]. Society is, thus, grappling with an escalating demand for innovative solutions that can effectively support the mounting needs of this burgeoning demographic. As a result, the last few decades have witnessed a swift rise in the deployment of smart aged-care products [2] or social service robots [3], particularly in the healthcare sector, as a means to meet this demand. Prominent companion robots, like Paro, not only assist but also provide emotional support, improving the quality of life of elderly people [4,5]. These robots serve not merely as assistive tools, but as companions that can provide emotional support, thus opening up a new frontier in human–robot interaction (HRI). While companion robots show potential in healthcare, understanding their successful interaction with the elderly, who may heavily rely on them, remains a challenge. The HRI field is presently a focal point of exhaustive research as scientists aim to deepen our understanding of the myriad facets that define and influence this complex interplay. Unraveling the root causes of discomfort or mistrust during interactions is essential for fostering deeper affinity and trust toward robots as social partners [6].

One crucial component that has been highlighted in these studies is the role of empathy, a human capacity that is pivotal to understanding the emotional and mental states of others [7]. As innovative humanoid robots, like Ameca, are widening the emotional bandwidth, it is important to bridge the human–robot social divide with a broader range of emotions [8]. Thus, empathy is seen as a key factor determining the success of these interactions [9], which is often delineated into two aspects, affective and cognitive [10]. Affective empathy concerns the ability to resonate emotionally with others, while cognitive empathy involves understanding others’ thoughts and emotions, a process that often necessitates perspective-taking [11]. Recent research has elucidated a nuanced relationship between empathy and perceptual face recognition skills. For instance, perspective-taking, a sub-component of cognitive empathy, has been linked to the accuracy of recognizing and dismissing certain emotional faces. Furthermore, perspective-taking is related to expedited reaction times when discarding faces expressing disgust [12]. Hence, cognitive empathy offers profound insights into the processes and mechanisms that engender meaningful engagement, thereby contributing to the design of robots that are adept at addressing user needs and adapting to an array of social contexts.

While cognitive empathy in HRI is less explored, its role in perceiving a robot’s life-likeness is significant. The role of a robot’s animacy in influencing individuals’ cognitive empathy during HRI is a contested subject with inconsistent findings in research. On one hand, theories such as simulation theory [13] and group classification theory [14] suggest that a higher degree of robot animacy—meaning robots that more closely resemble humans—promotes greater perspective-taking, where individuals more readily adopt the robots’ perspectives. Supporting evidence has been furnished by researchers such as Amorim, Isableu, and Jarraya [15], who found that as an object’s animacy increased, participants became more proficient in reasoning about the object-centered perspective by employing self-centered analogies. Moreover, Carlson et al. [16] revealed that when the interactive partner is a robot, an individual tends to assume their own perspective rather than that of the robot. On the other hand, Mori’s uncanny valley theory [17,18] argues that high-animacy robots may cause decreased familiarity and emotional distance, leading to reduced perspective-taking. For example, Yu and Zacks [19] discovered that human-like visual stimuli are more likely to elicit a person’s self-centered perspective, while inanimate objects are more inclined to be regarded from their own perspective. Similarly, Zhao, Cusimano, and Malle [20] found that people tend to adopt a robot’s perspective rather than that of a human-like entity when the robot displays nonverbal behaviors. Meanwhile, some recent studies challenge both perspectives, suggesting that an individual’s perspective-taking does not change regardless of whether they interact with a robot or a human [21]. Given these conflicting views, further research is needed to clarify the impact of robot animacy on cognitive empathy during HRI.

Our study explores the interplay between robot animacy, facial expressions, and participant’s age, aiming for holistic insight. While individual studies have touched upon these elements in isolation, our integrative approach seeks to provide a nuanced understanding that could guide future designs and strategies in the realm of HRI. By deciphering the synergies and conflicts among these variables, we aspire to set a new benchmark in designing robots that can seamlessly fit into the healthcare needs of the aging population. The equation becomes even more complex when considering the influence of positive facial expressions and age. Prior research has elucidated that positive facial emotional expressions possess the capacity to augment cognitive empathy [22]. Investigations have revealed that robot visages adorned with happy facial expressions are perceived as more animate than their neutral counterparts [23]. Another study discovered a correlation between facial emotional expression and animacy perception, with robots exhibiting joyous expressions more likely to be perceived as possessing cognition compared to those bearing neutral expressions [24]. Additionally, neuroimaging evidence indicates that positive social–emotional text stimuli can activate brain regions associated with adopting a third-person perspective, thereby bolstering perspective-taking abilities [25]. However, to the best of our knowledge, no previous research has explored whether positive facial emotional expressions can improve animacy perception and further enhance perspective-taking abilities, which holds significant implications within the realm of HRI.

Age greatly influences perspective-taking abilities, which decline with advancing individuals [26,27], possibly due to reduced activity in brain regions associated with tasks that differentiate between self and others’ perspectives [28,29]. Adding to this discourse, a recent study identified that adults maintain a consistent performance on the director task (DT), a referential-communication measure of perspective-taking, up until their late 30s; thereafter, a decline is observed, partially influenced by individual differences in executive functions [30]. Moreover, further studies have revealed that emotion and animacy can influence the perspective-taking performance of older individuals. In accordance with socio-emotional selectivity theory, older adults exhibit a preference for attending to positive emotions [31]. Additionally, a separate study indicated that older adults are less inclined to observe low-animacy robots [32]. However, previous research has not delved into how perceiving animacy and emotional expressions affects perspective-taking abilities in older adults.

This study addresses the above gaps by investigating how animacy, emotional facial expressions, and age impact perspective-taking in HRI. Our hypotheses are as follows: (1)The more human-like a robot is, the better its perspective-taking abilities (simulation and group classification theories, and (2)positive emotional expressions displayed by a robot will amplify perspective-taking abilities compared to neutral ones, considering the social significance of positive emotions. We will test these hypotheses by employing the DT to assess perspective-taking abilities across different age groups, degrees of animacy, and emotional expressions. The experimental results expand and support the group classification theory, offering explanations for the conflicting results observed in the relationship between cognitive empathy and anthropomorphism. Our research is the first to incorporate both age and expression variables, unearthing a unique finding that elderly individuals show a preference for high-anthropomorphic robots while being repelled by low-anthropomorphic robots. Significantly, positive expressions further intensify these inclinations (a discovery that was previously overlooked); this carries paramount implications for designing companion robots for the elderly.

## 2. Materials and Methods

### 2.1. Determination of the Sample Size

In this study, we used the G*Power 3.1 software [33] to calculate the optimal sample size, ensuring robust and reliable results. Considering an effect size of 0.25, an alpha value of 0.05, and aiming for a power (1-beta) of 0.8 [34], we identified the need for a sample size of 20. Our investigation involved two distinct groups, each subjected to six measurements over a defined period of time. This longitudinal approach allowed us to capture the dynamics of the phenomenon under examination comprehensively. Furthermore, we accounted for the correlation between repeated measures, which was estimated at 0.5, indicating a substantial association among the variables across different measurement instances. Applying these parameters within the G*Power 3.1 software, we calculated that a minimum sample size of 20 participants was necessary to reliably detect the significant effects. This determination ensures that our study possesses adequate statistical power to discern the hypothesized relationships accurately.

### 2.2. Participants

Our study involved 30 older adults, 16 males, with a mean age of 67.40 years (SD = 5.94 years) and an average education level of 9.63 years. They were recruited from a residential estate in Anhui, China. A public announcement was placed in the community center, inviting elderly individuals to participate in our study. Interested individuals underwent an initial phone screening for eligibility and were provided with comprehensive details about the study once deemed eligible. Additionally, 30 young adults, including 14 males, also participated in the study. The younger cohort, with an average age of 19.68 years (SD = 1.18 years) and an average education level of 15.47 years, were recruited from Southwest University. Announcements inviting participation were made via campus noticeboards and classroom announcements, and potential participants could register through a designated portal. Subsequent screenings determined their final eligibility. These demographics are summarized in Table 1.

To ensure the validity of our findings, we established strict eligibility criteria. Every participant was mandated to possess normal or corrected-to-normal vision and demonstrate proficiency with their dominant hand. For older adults, the Chinese versions of the mini-mental state examination (MMSE) [35] were employed. This instrument verifies the cognitive health of participants, especially given our study’s emphasis on perspective-taking capabilities, which can decline with age. Participants showing scores indicative of cognitive impairment were excluded from the study, in alignment with existing literature [36,37]. Moreover, all participants underwent an evaluation using the Toronto Alexithymia Scale-20 (TAS-20) [38]. This assessment was chosen to screen participants who might have difficulty identifying and expressing emotions, an essential criterion considering our study’s focus on robot facial expressions. Individuals demonstrating signs of alexithymia, based on established benchmarks [39,40], were not included in the final participant pool.

The recruitment of participants and experimental procedures were approved by the Ethics Committee, Faculty of Psychology of Southwest University (NO. IRB-H23105). Following ethical standards, we obtained informed consent from all participants. They were given a thorough explanation of the study’s aims, procedures, and implications. Post-experiment, a debriefing session was held to discuss the study’s design, outcomes, and significance, fostering an enriching exchange of knowledge. To show our appreciation for their participation, each participant received a token of appreciation worth CNY 30 Their involvement was instrumental in our pursuit of scientific knowledge.

### 2.3. Materials and Procedure

**Figure 1:**
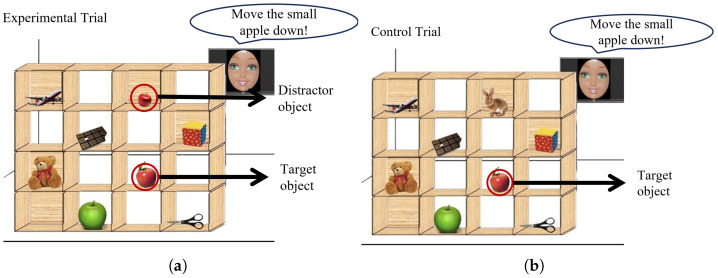
Examples of stimuli used in the director task (DT). (**a**) The experimental trials demonstrate scenarios where the participant is given privileged access to a distractor object, distinct from the target object. (**b**) The control trials (C2), on the other hand, showcase situations where both the target and competitor objects are equally accessible, thereby presenting a shared perspective for both objects. Accompanying the visual stimuli are AI-generated prompts, such as “Please move the small apple down”.

The DT, originally developed by Santiesteban et al. [41], was adapted to better fit the context of our study. Central to our adaptation was the replacement of the director; instead of the original Caucasian middle-aged male, we introduced a robot face with varying degrees of animacy, expressions, and verbal instructions, isolating factors on the specific nuances of facial features. In this meticulously designed experiment, a visually captivating 4 × 4 grid shelf filled with a diverse array of intriguing objects (as shown in Figure 1) graced the center stage of the computer screen. The display was a 15.6-inch monitor with a resolution of 1920 × 1080 pixels and a display area of 348 × 190 mm, located approximately 45 cm from the participant. A director, placed behind the shelves in the visual stimuli, provided instructions about moving objects for each trial. Participants were required to follow the verbal instructions generated by artificial intelligence (AI) from the director, moving objects on the shelf while considering the director’s perspective throughout each trial.

The DT included an experimental condition accompanied by two control conditions (C1 and C2), enhancing the robustness of the study. In the experimental condition (E), a conflict arose between the perspectives of the participants and the director. For example, when presented with a scenario like the one shown in Figure 1a and asked to “move the small apple left”, participants faced a predicament. They needed to disregard the smaller apple visible from their vantage point—hidden from the director due to the shelf’s obstructive backboard—and instead select the next smallest apple that the director could see. This conflicting perspective added to the task’s complexity and required keen decision-making skills from the participants.

On the other hand, control condition C1 did not present any conflict between the participant and the director. In this condition, the director simply instructed participants to move a non-conflicting item to a clear spot on the grid, such as “Move the chocolate left”. This condition served as a baseline, assessing the participant’s ability to follow clear instructions without conflict. Control condition C2 followed a similar pattern as the experimental condition (E), but the conflicting object in E was replaced with a completely unrelated item, as shown in Figure 1b. The instruction, however, remained unchanged. This cleverly designed condition aimed to isolate and examine the impact of the conflicting object in E, allowing researchers to understand its specific influence on participants’ performance.

The participants’ responses were meticulously recorded and graded based on a scoring system derived from [42]. Each response received a score indicating the level of accuracy exhibited, where a correct selection and subsequent precise movement merited a score of 1. A partially accurate selection paired with an incorrect movement garnered a score of 0.5, while an erroneous selection warranted a score of 0. This rigorous scoring method ensured that the participants’ performance was objectively evaluated, adding robustness to the study’s findings. To comprehensively fulfill the research objectives of this study, the director image underwent a remarkable transformation, giving way to facial visual stimuli possessing varying degrees of animacy and emotional expressions. These captivating visuals were meticulously crafted using FantaMorph software, which skillfully merged dolls and human faces. The original facial stimuli images were sourced from the esteemed image database meticulously curated by [23].

**Figure 2:**
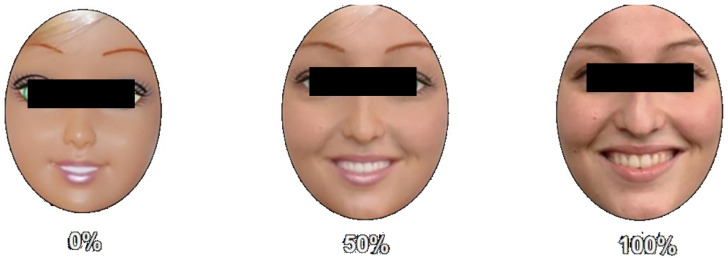
Examples of facial stimuli used in the DT, which shows a set of female face stimuli, each exhibiting happy expressions, but with differing degrees of animacy: 0%, 50%, and 100%.

In this experiment, we sought to address the limitations of existing research in the field of HRI by adopting a novel and comprehensive approach to investigate the relationship between animacy and cognitive empathy. Unlike prior studies that primarily focused on binary comparisons (e.g., high- versus low-animacy robots [43,44,45], and humans versus humanoid robots [19,20,21], we designed a more intricate experimental setup that encompassed a rich tapestry of stimuli variations. For instance, Złotowski et al. (2016) [43] primarily focused on how the appearance of a robot affects the perceived trustworthiness and empathy based on its behavior, mainly contrasting cartoonish figures against highly human-like figures. While their research offered crucial insights, it catered to a binary understanding. In contrast, our experiment delved deeper, moving beyond binary contrasts and embracing a multi-faceted approach. The face stimuli images featured three different levels of animacy (0%, 50%, 100%), infused with two distinct emotional states (happy and neutral), and belonging to both male and female genders. This intricate fusion resulted in a total of 12 experimental conditions, each encapsulating a unique combination of stimuli elements (as illustrated in Figure 2). The inclusion of both male and female faces within each experimental condition effectively nullified any potential own-gender bias, aligning with the meticulous methodology employed in this study, as exemplified by [46]. Each image boasted a resolution of 243 × 335 pixels, ensuring visual clarity and precision throughout the experiment.

The entire experiment unfolded across 12 blocks, featuring a grand total of 144 meticulously intertwined trials, presented in a carefully constructed pseudo-random order. To ensure that participants were suitably prepared, a brief four-trial practice session preceded the main experiment, offering an opportunity for familiarization with the task’s intricacies and optimizing performance during the subsequent trials.

## 3. Results

Eight young and five older participants scored > 61 on the TAS-20, and their datasets were excluded from data analysis. No older participant was excluded on the basis of the MMSE scores. After the exclusion, 25 young (67.40 ± 1.15 years, 9 males) and 22 older (21.48 ± 6.27 years, 14 males) participants were included in the data analysis. In alignment with the objectives of our research, we predominantly focused on the scores under the ‘E’ condition, which we interpret as reflecting the perspective-taking ability. These scores, serving as our dependent variables, were subjected to a mixed-design ANOVA. This statistical analysis model included a three-level within-subject factor of animacy (0%, 50%, 100%), a two-level within-subject factor of emotion (neutral, happy), and a two-level between-subject factor of the participant’s age (young, older adult). The Bonferroni method was employed for all post hoc multiple comparisons.

### 3.1. Animacy Influence on Task Accuracy

**Figure 3:**
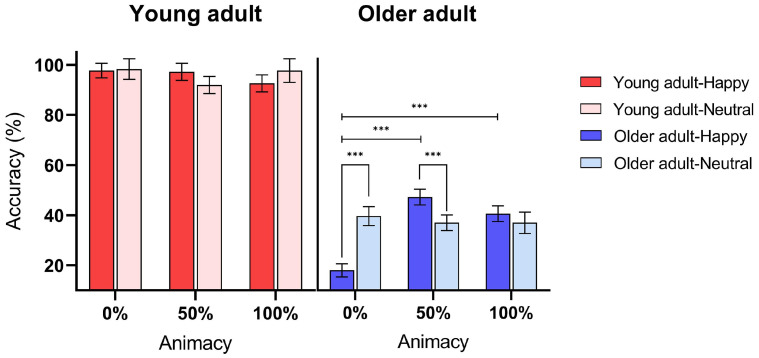
The accuracy of DT performance under varying levels of animacy and emotional stimuli in older adults (represented by blue bars) versus young adults (represented by red bars). Deeper-colored bars depict participants’ performance on trials featuring a happy facial expression (conveyed by the director), whereas lighter-colored bars illustrate the performance on trials with a neutral facial expression (conveyed by the director). Performance accuracy across all conditions was significantly lower in the older adult group compared to the young adult group. Error bars represent ± 1 mean standard error (MSE), and *** represents *p* < 0.001.

A significant main effect of age was observed (F[1,45] = 212.933, *p* < 0.001, $\eta_{p}^{2}$ = 0.826), indicating that the accuracy of the older adult group in the DT was significantly lower than that of the young adult group across all experimental conditions, as depicted in Figure 3. Concurrently, the main effect of animacy also manifested significantly (F[2,90] = 4.857, *p* = 0.010, $\eta_{p}^{2}$ = 0.097). Post hoc paired *t*-tests further revealed that participants’ overall DT accuracy under the 50% animacy condition was significantly higher than under the 0% animacy condition (t[44] = 3.40, *p* = 0.005, Cohen’s *d* = 1.03). Nevertheless, no significant difference was detected between 0% and 100% animacy conditions, or between 50% and 100% animacy conditions (detailed statistical results are provided in Appendix A Table A1). The main effect of emotion was not significant (F[1,45] = 0.966, *p* = 0.331, $\eta_{p}^{2}$ = 0.021). Our results clearly showcase the age-related differences in the director task (DT) performance. Older adults consistently performed with less accuracy across all experimental conditions, indicating a potential decline in their perspective-taking abilities with age.

**Figure 4:**
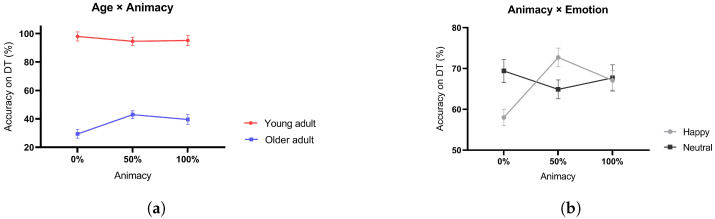
Comparative mean accuracy of the DT across (**a**) various levels of animacy stimuli for the two age groups, and (**b**) different levels of animacy for two emotional conditions. Error bars denote ± 1 MSE.

Importantly, we noted a significant interaction effect between animacy and age (F[2,90] = 13.864, *p* < 0.001, $\eta_{p}^{2}$ = 0.236) (refer to Figure 4a). Further analysis revealed that older adults’ accuracy on the DT with 0% animacy stimuli was significantly lower than their accuracies on both 50% (t[44] = 6.476, *p* < 0.001) and 100% animacy stimuli (t[44] = 4.478, *p* < 0.001), suggesting that an intermediate level of human-likeness in robots might be the most comprehensible or relatable for participants. No significant difference was discerned between 50% animacy vs. 100% animacy conditions (*p* = 0.558), indicating that extreme levels of animacy (fully robotic or fully human-like) may not drastically differ in their impact on perspective-taking abilities. Such a difference was not observed in the group of young adults (refer to Table A2). Additionally, across all three animacy conditions, the DT performance of the older adult group was significantly poorer than that of the young adult group (0% animacy: t[44] = 15.591, *p* < 0.001; 50% animacy: t[44] = 13.231, *p* < 0.001; and 100% animacy: t[44] = 11.100, *p* < 0.001, refer to Table A3 for detail information).

### 3.2. Interplay of Emotion and Animacy on Task Performance

We observed a significant interaction between animacy and emotion (F[2,90] = 17.976, *p* < 0.001, $\eta_{p}^{2}$ = 0.285) (see Figure 4b). Post hoc analysis showed that under the happy emotion condition, participants’ DT accuracy on 0% animacy condition was significantly lower than both 50% animacy (t[44] = 7.000, *p* < 0.001) and 100% animacy (t[44] = 3.792, *p* < 0.001) conditions. However, no significant difference was found between the 50% animacy vs. 100% animacy (*p* = 0.098) conditions. Under the neutral emotion condition, no such difference can be found (refer to Table A4). In the 0% animacy condition, participants’ accuracy under the happy emotion condition was significantly lower than the neutral emotion condition (t[44] = 5.700, *p* < 0.001). In the 50% animacy condition, the accuracy under the happy emotion condition was significantly higher than the neutral emotion condition (t[44] = 3.250, *p* = 0.003). In the 100% animacy condition, no such difference can be found (*p* = 0.791) (refer to Table A5). These results might suggest that the emotion of happiness serves as a reinforcement mechanism in older adults, specifically strengthening their tendency to socially exclude out-group interactors (low animacy) and embrace in-group interactors (high animacy).

**Figure 5:**
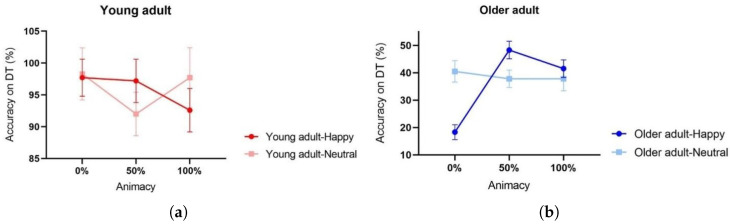
Mean DT accuracy for (**a**) young and (**b**) older adults across various levels of animacy and emotional expressions. Error bars denote ± 1 MSE.

We found no interaction effect between emotion and age (F(1, 45) = 0.966, *p* = 0.727, $\eta_{p}^{2}$ = 0.399). However, the interaction between animacy, emotion, and age was significant (F[2,90] = 13.552, *p* < 0.001, $\eta_{p}^{2}$ = 0.231) (see Figure 5). Post hoc analyses revealed that for the happy emotion condition, older adults’ DT accuracies on 0% animacy stimuli were significantly lower than 50% (t[44] = 10.345, *p* < 0.001) and 100% animacy (t[44] = 7.030, *p* < 0.001) conditions. No significant difference can be found between 50% vs. 100% animacy conditions (*p* = 0.182) (refer to Table A6).

In the 0% animacy condition, older adults’ accuracy on the neutral emotion was significantly lower than the happy emotion (t[44] = 7.929, *p* < 0.001). However, in the 50% animacy condition, the neutral expression accuracy was significantly higher than happy (t[44] = 3.182, *p* = 0.003). In the 100% animacy condition, we found no significant difference between the two emotion conditions (*p* = 0.290) (refer to Table A7). In addition, we found significant age differences in accuracy across different levels of animacy and emotion conditions.

## 4. Discussion

This study was designed to fill a notable research gap in the field of HRI by exploring how the varying levels of facial animacy and emotional facial expressions can together influence perspective-taking abilities in different age groups, with a particular emphasis on older adults. This research carries special relevance as it pertains to caregiving scenarios for older adults, where robots may play a role in providing daily assistance and emotional interaction. Contrary to our initial predictions, our findings indicated that perspective-taking was notably more challenging for older adults when interacting with low-animacy robot-like faces, compared to high-animacy human-like faces. Additionally, when the low-animacy interactors displayed happy facial expressions, there was a surprising decrease in the older adults’ perspective-taking abilities, compared to interactors with neutral expressions.

### 4.1. Preference for High Animacy in Older Adults

Our findings suggest that older adults display a pronounced preference for high-animacy faces (50% & 100%) over low-animacy faces (0%) in terms of perspective-taking. This contrast was not statistically significant for young adults across different animacy levels (0% vs. 50% vs. 100%), a phenomenon that could potentially be explained by a ceiling effect.

Notably, the observed outcomes among the older population are more closely aligned with the group classification theory than the simulation theory. Contrary to our initial hypothesis, which was grounded in simulation theory, the perspective-taking of older adults did not incrementally increase with animacy. Instead, their responses to faces with higher animacy levels (50% vs. 100%) showed no significant difference. This implies that once facial animacy surpasses a certain threshold (roughly 50% in this study), older adults are likely to classify it as an in-group member, thus exhibiting greater perspective-taking toward it compared to out-group faces with 0% animacy. This finding can potentially be rationalized by the fact that older adults have had fewer interactions with low-animacy objects. During their formative years, low-animacy items, such as robots and cartoons, were not as ubiquitous as they are in contemporary society. Humanoid robots did not garner global public recognition until the 19th century [47], and they arrived even later in China. As a result, in social interaction scenarios, older adults may perceive low-animacy objects as out-group members with which they maintain a mental distance [48]. They may experience difficulty in achieving cognitive empathy with low-animacy objects, viewing them as unfamiliar, and possibly threatening [49].

Another lens through which to interpret these results is the socio-emotional selectivity theory. This theory posits that older adults, aware of their limited lifespans, are likely to prioritize meaningful emotional regulation [50]. Therefore, if these low-animacy faces cannot provide them with sufficient emotional support or meet their emotional needs, older adults may reduce their interactions with them [17]. This situation can also be observed in their daily lives; for example, they may prefer to interact with humans or pets rather than robots or other humanoid objects.

### 4.2. Reinforcing the Effect of Emotion

Our findings indicate that as the facial animacies of interactors decrease, older adults exhibit reduced perspective-taking abilities with happy expressions compared to neutral ones. However, when facial animacy rises above 50%, their perspective-taking abilities show a significant increase with happy expressions compared to neutral ones. This counters our initial hypothesis that happiness would lead to enhanced perspective-taking abilities. These results might indicate that the emotion of happiness serves as a reinforcement mechanism in older adults, specifically strengthening their tendency to socially exclude out-group interactors (low animacy) and embrace in-group interactors (high animacy). These findings build upon existing literature [51,52], indicating that when positive expressions are perceived as ‘false smiles’ or ‘strange’, they can hinder rather than aid the establishment of connections and cognitive empathy. Additionally, neuroscience research provides insights that reinforce the impact of happiness on in-group favoritism [53]. Specifically, mirror neurons play a significant role, ‘mirroring’ others’ emotions within one’s neural circuit [54]. In-group faces displaying happiness elicited more robust mirror neuron emotional responses [55]. These results consolidate our understanding of the reinforcing effect of happiness on in-group faces.

These results have significant implications for the design of robots and AI, especially those aimed at interacting with older adults. As the field of HRI continues to evolve, it will be crucial to consider how subtle cues in facial expressions can profoundly impact a user’s sense of comfort and willingness to engage with these entities. In particular, in cultures where emotional restraint is valued, such as many Asian cultures, the importance of nuanced and genuine facial cues should not be underestimated [56]. When designing robots that can effectively interact with different cultural groups, the animacy and emotional authenticity of facial expressions should be carefully calibrated to optimize user engagement and acceptance.

### 4.3. Other Effects, Limitations, and Prospects

With respect to young adults, there was no significant DT accuracy difference between different experimental conditions. We assumed that it was caused by the ceiling effect of young adults. It has been suggested that DT might be too simple for young adults with significantly faster response speeds and more robust executive functions than older adults. The high accuracy rate of DT in young adults was also found in [30]. It was found that young adults aged 20–27 years made significantly fewer errors in DT than those over 38 years old. This was also reflected in a similar study [57]. Moreover, past evidence has consistently reported the presence of the uncanny valley effect in young adults [17]. This effect suggests that as robots become more human-like, they elicit positive attitudes until a point of close resemblance is reached, beyond which sentiment becomes sharply negative, before improving with further human likeness. Thus, it should be further explored why such a difference cannot be observed in this study. Moreover, when considering the perspective-taking of older adults, the existing literature has suggested that cognitive empathy and perspective-taking abilities may decline with age [29,58]. In line with these findings, our study observed a similar trend using this unique research paradigm. However, the complexities of these mechanisms and their interactions with factors such as animacy necessitate further research, not only to confirm the implications of these differences but also to elucidate the underlying mechanisms driving these changes.

Additionally, the results did not prove the uncanny valley effect, which might be due to two reasons. The first possible reason is that the uncanny valley effect may be more involved in emotional evoke, and more related to affective empathy [59,60]. However, perspective-taking was more related to cognitive empathy [61]. The results suggested that this emotional evoke could not affect the cognitive process. The second possible reason is due to the lack of continuous animacy materials in the study. In this study, only 0%, 50%, and 100% animacies were used due to limited resources.

Several limitations are involved in the current study. Firstly, the face stimuli used in this study are of Caucasians and young adults, which may potentially introduce confounding variables such as the other-race effect [24] and own-age bias [62]. Moreover, the participant sampling predominantly involved cognitively healthy and highly educated older adults, which may limit the generalizability of the results to a broader elderly population, particularly those suffering from cognitive impairments, such as dementia. Secondly, the complexity of HRI may have been overlooked in this study, especially considering that experiments conducted in lab settings may not fully simulate the complexity of real-world scenarios or long-term usage. Furthermore, the study did not delve into the intricate mechanisms of cognitive empathy, a complex psychological process involving understanding and perceiving the emotions and perspectives of others. It might be challenging to gain deep insights into the underlying mechanisms by merely observing surface indicators, like facial expressions. These factors may have limited the applicability and depth of this study to some extent.

## 5. Conclusions

This study illuminates the substantial impact of facial animacy and emotional expression on the perspective-taking abilities of older adults, offering profound insights into HRI and cognitive empathy. The results suggest that robot faces with high animacy and happy expressions are more likely to elicit effective perspective-taking among the elderly. From a methodological standpoint, our innovative blend has allowed for a more nuanced and detailed understanding of cognitive empathy and perspective-taking in HRI. This breakthrough approach helped bridge an essential knowledge gap, offering a comprehensive perspective on how age, facial animacy, and emotional expressions influence cognitive empathy. Furthermore, these findings hold critical implications for the design of social service robots, particularly those targeting an older demographic. When designing AI and robot interactions for older adults, the influence of animacy and emotional expression should be considered and adjusted according to the specific needs and preferences of the elderly. This is vital for optimizing the user experiences for older adults, enhancing their acceptance of robots, and ultimately improving their quality of life.

Investigating human–robot interactions in real-world settings, such as homes or healthcare facilities, is a key next step, given our study’s controlled environment. The influence of everyday contexts on these interactions is crucial to understand. Moreover, as AI advances, studying the effects of enhanced algorithms on humanoid robots and their impact on the perceptions of older adults is essential. Such research aims to fine-tune interactions to suit the specific needs of the elderly.

## Figures and Tables

**Table 1:** Demographics of participants.

	Older Adult	Young Adult
Male	16	14
Female	14	16
Age	60–83	18–22
	M = 67.40	M = 19.68
	SD = 5.94	SD = 1.18
Level of education	9.63	15.47
Total number	30	30

## Data Availability

The datasets used and/or analyzed during the current study are available from the corresponding author upon reasonable request.

## Abbreviations

The following abbreviations are used in this manuscript:

AI	artificial intelligence
DT	director task
HRI	human–robot interaction
MMSE	mini-mental state examination
TAS-20	Toronto Alexithymia Scale-20

## Appendix A

**Table A1:** Post hoc multiple comparisons examining the impact of animacy on director task (DT) accuracy, using Bonferroni corrections; *p*-values achieving statistical significance are marked (*).

Animacy Level	M	SD	df	t	*p*	Cohen’s *d*
0	0.637	0.022	44	−3.40	0.005 *	1.03
50	0.688	0.020				
0	0.637	0.022	44	2.18	0.106	0.66
100	0.674	0.025				
50	0.688	0.020	44	0.78	1.000	0.23
100	0.674	0.025				

## Appendix B

**Table A2:** Post hoc multiple comparisons examining the impact of animacy on DT accuracy for different age groups. *p*-values achieving statistical significance are marked (*).

Age	Animacy Level	M	SD	df	t	*p*	Cohen’s *d*
Youth	0	0.980	0.032	44	1.545	0.395	0.466
	50	0.946	0.029				
	0	0.980	0.032	44	1.120	0.780	0.338
	100	0.952	0.036				
	50	0.946	0.029	44	0.222	1.000	0.067
	100	0.952	0.036				
Elderly	0	0.294	0.030	44	6.476	<0.001 *	1.922
	50	0.430	0.027				
	0	0.294	0.030	44	4.478	<0.001 *	1.350
	100	0.396	0.034				
	50	0.430	0.027	44	1.360	0.558	0.410
	100	0.396	0.034				

**Table A3:** Post hoc multiple comparisons examining the impacts of different age groups (youth vs. elderly) on DT accuracy across animacy levels. *p*-values achieving statistical significance are marked (*).

Animacy Level	Age	M	SD	df	t	*p*	Cohen’s *d*
0	Youth	0.980	0.032	44	15.591	<0.001 *	4.701
	Elderly	0.294	0.030				
50	Youth	0.946	0.029	44	13.231	<0.001 *	3.989
	Elderly	0.430	0.027				
100	Youth	0.952	0.036	44	11.100	<0.001 *	3.467
	Elderly	0.396	0.034				

**Table A4:** Post hoc multiple comparisons examining the impact of animacy on DT accuracy for different emotion conditions. *p*-values achieving statistical significance are marked (*).

Emotion	Animacy Level	M	SD	df	t	*p*	Cohen’s *d*
Happy	0	0.580	0.020	44	7.000	<0.001 *	2.111
	50	0.727	0.023				
	0	0.580	0.020	44	3.792	0.001 *	1.143
	100	0.671	0.024				
	50	0.727	0.023	44	2.154	0.098	0.649
	100	0.671	0.024				
Neutral	0	0.694	0.028	44	2.143	0.111	0.646
	50	0.649	0.023				
	0	0.694	0.028	44	0.773	1.000	0.233
	100	0.677	0.032				
	50	0.649	0.023	44	1.077	0.816	0.325
	100	0.677	0.032				

**Table A5:** Post hoc multiple comparisons examining the impacts of different emotion conditions (happy vs. neutral) on DT accuracy across animacy levels. *p*-values achieving statistical significance are marked (*).

Animacy Level	Emotion	M	SD	df	t	*p*	Cohen’s *d*
0	Happy	0.580	0.020	44	5.700	<0.001 *	1.719
	Neutral	0.694	0.028				
50	Happy	0.727	0.023	44	3.250	0.003 *	0.980
	Neutral	0.649	0.023				
100	Happy	0.671	0.024	44	0.269	0.791	0.081
	Neutral	0.677	0.032				

## Appendix C

**Table A6:** Post hoc analysis examining the impact of animacy conditions across different emotions on DT accuracy for different age groups. *p*-values achieving statistical significance are marked (*).

Age	Emotion	Animacy Level	M	SD	df	t	*p*	Cohen’s *d*
Youth	Happy	0	0.977	0.029	44	0.194	1.000	0.058
		50	0.972	0.034				
		0	0.977	0.029	44	1.457	0.449	0.439
		100	0.926	0.034				
		50	0.972	0.034	44	1.216	0.691	0.367
		100	0.926	0.034				
	Neutral	0	0.983	0.041	44	2.032	0.139	0.613
		50	0.920	0.034				
		0	0.983	0.041	44	0.182	1.000	0.055
		100	0.977	0.047				
		50	0.920	0.034	44	1.541	0.403	0.465
		100	0.977	0.047				
Elderly	Happy	0	0.183	0.027	44	10.345	<0.001 *	3.119
		50	0.483	0.032				
		0	0.183	0.027	44	7.030	<0.001 *	2.120
		100	0.415	0.032				
		50	0.483	0.032	44	1.914	0.182	0.577
		100	0.415	0.032				
	Neutral	0	0.405	0.039	44	0.931	1.000	0.281
		50	0.378	0.032				
		0	0.405	0.039	44	0.871	1.000	0.263
		100	0.378	0.044				
		50	0.378	0.032	44	0.000	1.000	0.000
		100	0.378	0.044				

**Table A7:** Post hoc analysis examining the impact of emotion across different animacy levels on DT accuracy for different age groups. *p*-values achieving statistical significance are marked (*).

Age	Animacy Level	Emotion	M	SD	df	t	*p*	Cohen’s *d*
Youth	0	Happy	0.977	0.029	44	0.200	0.849	0.060
		Neutral	0.983	0.041				
	50	Happy	0.972	0.034	44	1.417	0.157	0.427
		Neutral	0.920	0.034				
	100	Happy	0.926	0.034	44	1.378	0.178	0.415
		Neutral	0.977	0.047				
Elderly	0	Happy	0.183	0.027	44	7.929	<0.001 *	2.391
		Neutral	0.405	0.039				
	50	Happy	0.483	0.032	44	3.182	0.003 *	0.959
		Neutral	0.378	0.032				
	100	Happy	0.415	0.032	44	1.086	0.290	0.327
		Neutral	0.378	0.044				
